# Insensibility during Surgical Operations, Produced by Inhalation

**Published:** 1846-12

**Authors:** 


*Insensibility during Surgical operations, produced by inhalation.*—We had heard through a private source of a late discovery at Boston, Mass., by which teeth were extracted, and other severe surgical operations performed during a state of insensibility to pain, and partial unconsciousness, produced by inhaling a medicinal preparation. In the Boston Med. and Surg. Journal, Nov. 18th, we find a report on the subject, read before the Boston Society of medical improvement, by Henry Jacob Bigelow, M. D., one of the surgeons of the Mass. General Hospital. Several operations have been performed at the Hospital by Drs. Warren and Hayward, apparently without any, or with trifling pain. Dr. Bigelow states that no doubts existed in the minds of those who witnessed the operations that the unconsciousness was real, nor could the result be attributed in any degree to the imagination. Dr. B. thinks it promises to be one of the important discoveries of the age. In our next No. we will give extracts from the report presenting the details of the cases in which the experiments were made. The invention has been patented in the names of Dr. Charles T. Jackson, the distinguished chemist, and Dr. Morton, dentist, of Boston. Coming as the facts do, from a source which renders their authenticity unquestionable, they will be regarded with great interest by the profession and public. Assuredly the discovery of a perfectly safe method of divesting operations of pain, would constitute an epoch in the history of medicine, scarcely less prominent than that of vaccination by Jenner.

